# Our Contemporaries

**Published:** 1917-06

**Authors:** 


### OUR CONTEMPORARIES

The Society of Bacteriologists has undertaken to publish a new journal, Abstracts of Bacteriology, under the editorship of Dr. A. Parker Hitchens and Dr. George H. Smith of Glenolden, Pa.

"In our man-made world, the sexual exploitation of women as breeding machines is one of the saddest of our records." Medical Times. In what different persons regard, respectively as a God-made or a naturally evolved world, women might be considered as breeding machines. The protest against this fact comes several thousand or million years too late. In so far as the world may now be considered man-made, the saddest thing is the sexual exploitation of women—or of men by women—with the attempt to eliminate the latter as breeding machines.

